# Hepatic vessels segmentation using deep learning and preprocessing enhancement

**DOI:** 10.1002/acm2.13966

**Published:** 2023-03-18

**Authors:** Omar Ibrahim Alirr, Ashrani Aizzuddin Abd Rahni

**Affiliations:** ^1^ College of Engineering and Technology American University of the Middle East Egaila Kuwait; ^2^ Department of Electrical Electronic and Systems Engineering Faculty of Engineering and Built Environment Universiti Kebangsaan Bangi Selangor Malaysia

**Keywords:** abdominal CT, CED, deep learning, residual block, U‐net, vasculature segmentation

## Abstract

**Purpose:**

Liver hepatic vessels segmentation is a crucial step for the diagnosis process in patients with hepatic diseases. Segmentation of liver vessels helps to study the liver internal segmental anatomy that helps in the preoperative planning of surgical treatment.

**Methods:**

Recently, the convolutional neural networks (CNN) have been proved to be efficient for the task of medical image segmentation. The paper proposes an automatic deep learning‐based system for liver hepatic vessels segmentation of Computed Tomography (CT) datasets from different sources. The proposed work focuses on the combination of different steps; it starts by a preprocessing step to improve the vessels appearance within the liver region of interest in the CT scans. Coherence enhancing diffusion filtering (CED) and vesselness filtering methods are used to improve vessels contrast and intensity homogeneity. The proposed U‐net based network architecture is implemented with modified residual block to include concatenation skip connection. The effect of enhancement using filtering step was studied. Also, the effect of data mismatch used in training and validation is studied.

**Results:**

The proposed method is evaluated using many CT datasets. Dice similarity coefficient (DSC) is used to evaluate the method. The average DSC score achieved a score 79%.

**Conclusions:**

The proposed approach succeeded to segment liver vasculature from the liver envelope accurately, which makes it as potential tool for clinical preoperative planning.

## 1 INTRODUCTION

In several clinical applications, such as liver surgical resections and living donor liver transplantations, the segmentation of the hepatic vasculature from CT scans is an essential step for hepatic diagnosis (LDLT). Given the liver's ability to regenerate, surgical excision is a generally accepted treatment for metastases that develop inside the organ. Segmenting the liver's vasculature is a crucial first step in defining the segmental architecture for planning liver resection. However, the tumor's size, location, and link to the liver's vascular system all affect how easily it can be removed. It must be ensured that the liver tissues stay fed by the vascular system because tumors close to the vessels may not be amenable to surgery. This necessitates thorough familiarity with the liver's internal tumors and hepatic vascular organization. In order to ensure the safety of the donor and the patient's liver's functionality, the liver vascular anatomy must be precisely analyzed as part of the LDLT procedures.^1^, ^2^

Generally speaking, a skilled radiologist can manually segment the hepatic vasculature by drawing lines to indicate the locations of the veins in each CT scan slice. This task, however, is arduous, time‐consuming, and biased toward specialist knowledge. The automatic vessels segmentation thus garners considerable scientific interest. The technique of automatically separating the hepatic veins from the rest of the liver tissue is not simple and is fraught with difficulties. These difficulties include the intricacy of the vascular anatomy, anatomical variation from patient to patient, the presence of lesions, the low contrast of the arterial borders with the surrounding liver parenchyma, and the non‐uniform intensity values caused by contrast injection.^3^

The segmentation of the liver's vasculature is a crucial stage in computer‐aided systems used in liver surgery.^4^, ^5^ Vessel information extraction from 3D pictures has attracted a lot of attention due to its significance in numerous medical applications. For vessel segmentation, numerous segmentation methods have been suggested.^6^ However, because of the great structural differences, branching complexity, and tiny terminating vessel size in hepatic vessels, general vessels segmentation cannot be applied for hepatic vessels segmentation. Several techniques have been published for the segmentation of the hepatic vasculature.^2^

The goal of skeleton‐based approaches is to immediately locate blood vessel centerlines and then connect the centerlines to create the vessel tree; however, most of these techniques are interactive since it requires a lot of user involvement.^7^ An approach based on an interactive skeleton was proposed by Shen et al. It begins by thresholding the vessels to create a binary representation of the vascular structure. Then, by examining the local maximum voxels in the distance map of the vascular structure, extract the skeletonization.^8^ An approach by Soler et al. that uses anisotropic diffusion filtering to improve intensities and protect the vessel structures was put forth. To eliminate spurious branching based on topological properties taken from the built skeleton, they employed a thresholding technique followed by skeletonization.^9^

In region‐growing methods, all image voxels associated with blood arteries are grouped together based on a predetermined criterion, such as proximity or intensity similarity.^10^, ^11^, ^12^ Chi et al. suggested a method based on region growth that separates and segments the vasculature in the target CT scans by employing a context‐voting approach.^13^ Zeng et al. employed two separate techniques: active contour with k‐mean clustering for segmenting thick vessels and 3D region growth with Gaussian filtering for segmenting thin vessels.^14^ For the purpose of surgical planning, Beichel et al. proposed segmentation method that uses the portal veins to divide the liver into segments. They employed contrast‐enhanced data sets containing visible portal veins, and then they improved the veins using hessian methods.^15^ To demonstrate the impact of using various multiscale filtering approaches, Luu et al. combined the region growth method with several filtering strategies.^16^

One of the most utilized approaches for segmenting vessels is level‐set based deformable active contour methods since it is simpler to use them to extract specific forms and because they can adapt to complex object topologies.^17^ For the segmentation of vessels, Hong et al. used boundary information with region‐based level sets.^18^ To segment the hepatic vasculature, Jin et al. combined hessian‐based filtering with a level set active contour. To mitigate the effects of Gaussian filter blurring, the level set technique is updated to include the Gaussian standard deviation. In order to assess their approach, authors generated synthetic images.^19^

Deep fully convolutional networks (FCN) have recently been developed as a result of recent advancements in computer vision, which have improved the performance of semantic segmentation and enabled to outperform other competitors in the field of medical imaging.^20^, ^21^, ^22^ Convolutional neural networks (CNNs), a type of artificial neural network (ANN) designed for image processing, have emerged as the method of choice for vessel segmentation, including the hepatic vasculature, and have shown encouraging results in recent applications.^23^ The primary goal of the General FCN is image classification, where the input is an image and the output is a single label. However, the localization and segmentation of the area of vasculature are also necessary in the vessel's segmentation from chest CT analysis.^21^, ^24^ Deep neural network architectures for medical image segmentation have seen a significant change since U‐net first appeared.^25^ Residual convolution blocks, revised skip connections, dense convolution blocks, and attention techniques are now advantageous for many suggested U‐net based designs.^26^, ^27^

Ibragimov et al. used CNNs in order to segment the portal vein in liver CT images. They improved the outcomes using Markov random fields (MRF), which included deleting isolated regions brought on by segmentation mistakes. The detection of the portal veins centerline in accordance with anatomical characteristics of the PV, such as its branch composition and tubularity, was added to the results generated by CNN‐MRF‐based approaches.^28^ Automatic hepatic vascular segmentation based on a multi‐pathway CNN architecture was suggested by Kitrungrotsakul et al. Their architecture incorporates a variety of networks, each of which is designed to learn certain features from the processed vessel image. To fully extract the features, the learning process was applied to three planes, sagittal, coronal, and transverse.^29^ A multi‐task 3D fully convolutional neural network (3D‐FCN) was presented by Kehwani et al. for reconstructing the vessel tree.^30^ The suggested method estimates the boundaries in the rebuilt vessel tree and enables voxels to be found on vascular centerlines. A metric was established that accounts for both the topological distance inside each class of vascular pairs as well as the distance between classes. The shortest path tree algorithm and the newly learnt connectivity measure are then used to reconstruct vessel trees. Huang et al. used 3D‐U‐Net to extract liver blood vessels, they used several training samples and insufficient annotations. The settings are changed based on the ratio of successfully categorized foreground voxels to erroneously classified foreground voxels, using the Dice loss function, to improve segmentation outcomes in unbalanced classes. The penalty for improperly categorized voxels is also increased in order to train the network to identify vessels with weak borders, low contrast, and excessive noise.^31^

## 2 METHOD

The first preprocessing procedures included isotropic resampling to 1 mm^3^ resolution and extracting the liver ROI in the target CT scan's liver Region of Interest (ROI). The segmentation of the liver organ in this paper is accomplished using our previously suggested work that manages the task of segmenting the liver using CT scans.^32^ This paper presents a method for learning and extracting features of the liver vasculature from liver organ ROI using a deep learning methodology. The deep learning segmentation stage and vessel enhancement are the two key steps that make up the approach in general. The first stage is image pre‐processing, which tries to improve the look of the vasculature in the target hepatic region. The segmentation of the vasculature within the liver ROI faces a number of difficulties, including hazy margins, low contrast, and intensity inhomogeneity. Additionally, there is a lot of variation in the texture, size, and position of vascular regions in CT slices. The segmented liver (ROI) from the CT image is enhanced using two distinct filters, the vesselness filter and the tensor‐based coherence enhancing diffusion (CED) filtering, to increase the detection of the vessels.^33^ The liver's hepatic vessels must be segmented, with the exception of the inferior vena cava, which is not considered as a part of the liver vasculature.^34^ The main aim in this paper is to use of preprocessing filtering steps to enhance the training process of the U‐net based deep learning network. Also, the paper highlights the ResDense block as one of the residual blocks that improve the learning step.

### 2.1 Datasets

The Medical Segmentation Decathlon (MSD) is a large training dataset that is publicly available and is utilized in this article to train the FCN networks. This dataset, which contains vascular ground‐truths, has not yet been used in many study findings because it has only recently been published. The MSD library has 2633 3D images that were gathered from various sources, modalities, and anatomical regions of interest.^35^ The individuals in the Hepatic Vessels databases had a range of primary and metastatic liver tumors. There are 443 portal venous phase CT scans in the Hepatic Vessel task (Task08). Using the Scout app, the hepatic arteries were segmented semi‐automatically. Briefly, a level‐set based strategy was used to grow a seed point on the region of interest. Contours were manually adjusted by an expert abdominal radiologist.

### 2.2 Vessels appearance enhancement

Patient body motion and CT scanner noise are the two main causes of noise in CT scans. High resolution CT scans will be more susceptible to CT scanner noise. Since this noise has a significant impact on the structure of the vessels, it cannot be ignored. As a result, before beginning the segmentation of the vessels, a pre‐processing approach for vessel enhancement and noise filtering is created.

Either vesselness filters or noise reduction filters could be used to improve vessels. For the purpose of improving hepatic vasculature, Luu et al. compared the diffusion filters and vesselness filters quantitatively. They claimed that for big vessels with strong contrast and distinct boundaries, diffusion filters outperform vesselness filters. Additionally, it performs better at vessels bifurcations than vesselness filters, which struggle in situations like this. However, compared to diffusion filtering, which is based on smoothing and may cause the low contrast vessel to vanish, vesselness filters are better at enhancing the little low contrast vessels. Therefore, vesselness filtering works better for small, low‐contrast vessels while diffusion filters work better for large, high‐contrast vessels.^16^

In order to improve the appearance of the vessel structure in the target CT scans, a novel approach is proposed in this study that combines the vesselness enhancement and diffusion filtering techniques. The goal is to take advantage of these two strategies' advantages and get over their drawbacks.^16^ The Frangi vesselness filter, which is based on hessian theory, is used to strengthen tubular‐like structures while weakening non‐tubular ones. On the vesselness‐enhanced image, anisotropic coherence enhancing diffusion filtering (CED) is used. The coherence‐enhancing filter is used to denoise the mistakes and boost the coherence of the regions with comparable intensities while maintaining the vessels edges. The vesselsness filter attempts to enhance and extract the tube‐like structures.

#### 2.2.1 Vesselness enhancement

In portal venous phase CT scans, the hepatic and portal veins are contrast‐enhanced and appear substantially more intensely than the liver parenchyma. Some data sets, however, have a poor contrast to noise ratio. The vesselness filters are employed to improve and maintain the vessels' structural integrity. It has been utilized to enhance the tube‐like structure and inhibit other structures using a variety of vesselness augmentation filters. The Hessian‐based multi‐scale filtering used in this study, which is based on the work of Frangi,^36^ tries to distinguish between tubular‐like structures and blob‐ and plate‐like structures. The primary idea is that the images are convolved using 3D Gaussian filters at various scales, and the shape of the local structures within the images is determined by analyzing the eigenvalues of the Hessian matrix at each pixel or voxel in terms of a response function.^19^

To determine the maximum vesselness values, the Hessian enhancement is applied to the target image at various scales on each voxel. The contrast of the small vessels will be improved by the Hessian enhancement at small scale values (1–3), whereas the vessels with large radii will be enhanced at big scale values (4−8).^36^

The segmentation and separation of the vessels at their ends are the goals of this work. But because of the low resolution, it will be difficult to distinguish the ends of the vessels. In order to prevent improving such tiny connected ends, the vesselness is applied with large‐scale values (4−8) in the proposed vessel enhancement step. This ensures that the vessels are separated from one another so that we can separate them in the next steps.

#### 2.2.2 Anisotropic coherence enhancing diffusion filtering

The noise in the homogenous intensity zones must be reduced, and the smoothness must be improved, in order to improve the contrast in the target CT scans. Suppressing the artifacts while conserving and improving the vessels by retaining the contrast at their edges is necessary for better vessel extraction from CT scans. Anisotropic enhancing diffusion filtering shown its advantages over other filtering algorithms for this purpose.^33^ The scalar diffusion constant should be decreased at the steep edges of the linear diffusion filters in order to preserve the edge. However, this will produce noisy edges. In contrast, the anisotropic enhancing diffusion filters modified the diffusion along the image structures using the diffusion tensor. This diffusion filtering uses a structure tensor, which is based on the use of structure description such structure features or local coherence of structure, as a diffusion tensor to steer the diffusion.^37^

The edge enhancing diffusion (ED) and coherence enhancing diffusion (CED) filters are two proposed structure tensors by Mendrik et al. (EED). Compared to EED, CED is better suited to enhance tube‐like structures in pictures.^33^ Since CED is one‐dimensional diffusion, it may have diffusion in one dimension or none at all. It is developed to improve tube‐like structures. According to the structure tensor, the CED will have a diffusion in the direction of V3 if the (μ1 > μ2 > μ3 >) are the eigenvalues in the direction of the eigenvectors V1, V2, and V3 of the structure tensor. The performance of the diffusion will be determined by the ratio between the second and third eigenvalues. This ratio is high for structures that resemble vessels, whereas it is low for objects that like blobs or plates. It is intended to enhance the tube‐like structure and suppress the other plate‐ or blob‐like structures during the preprocessing stage. The smoothness of the diffusion along the vessel boundaries and the coherence of the intensities within the vessel areas are not guaranteed by vesselness, which could lead to unconnected vessels. To improve intensity coherence and smooth the tube‐like features, the coherence enhancing anisotropic diffusion filter, CED, is used.

**FIGURE 1:**
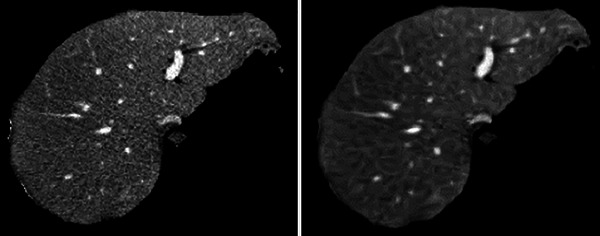
_(a) Original vessels appearance versus (b) enhanced vessels appearance._

During the preprocessing stage, vesselness is followed by CED, Figure 1 displays example of data set. The vessels in Figure 1b are more visible than the nearby liver tissue. It is evident that the CED filter was successful in enhancing diffusion along the regions of high intensity coherence, which is ideal for connecting the gaps between the vessels. Additionally, it lessens diffusivity along the irregularly shaped structures that preserve the distinct boundaries between the blood vessels and the liver parenchyma around them. This phase is thought to be a crucial requirement before using further vascular segmentation steps.

### 2.3 Training patches extraction

**FIGURE 2:**
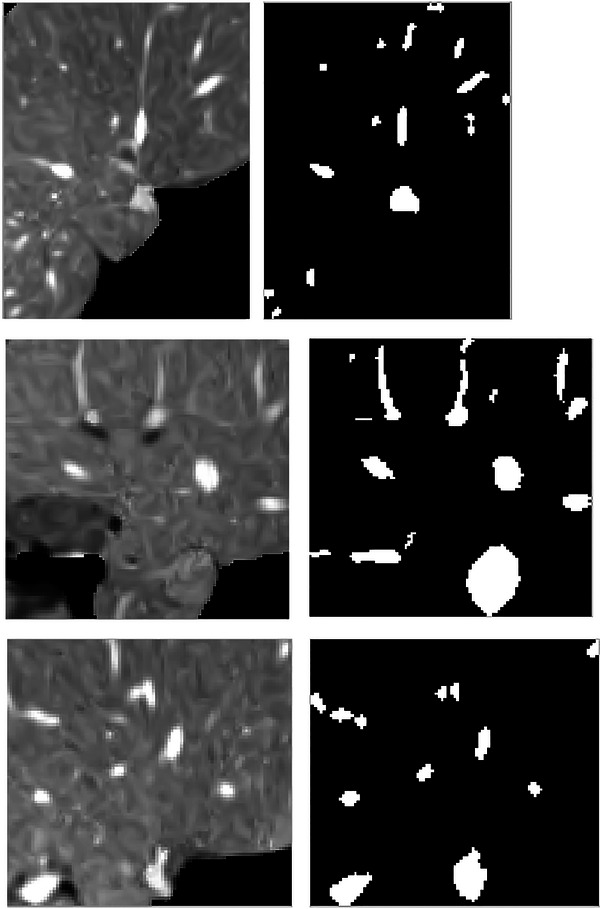
Different training patches with different sizes.

It is uncertain how the vessels are distributed and where they are located inside CT scans, which is regarded as the key worry for improving the suggested deep learning training. Additionally, if the CT slice has sections of vessels, the distribution within the slice is significantly skewed because only a tiny portion of the slice may be made up of vasculature. Therefore, a strong bias toward the background could result from utilizing the entire CT set as training patches, which is a common semantic segmentation issue in medical imaging. To solve this issue, the created training slices are randomly selected from the slices that have vessels in them using various patch sizes. The training datasets depict the liver slices that are solely taken from the ROI for the liver (not the whole CT slice). The generated patches have varying resolutions just as the liver organ appears in the various slices at various sizes. Examples of various training patches are shown in Figure 2.

### 2.4 Network architecture: ResDense FCN

**FIGURE 3:**
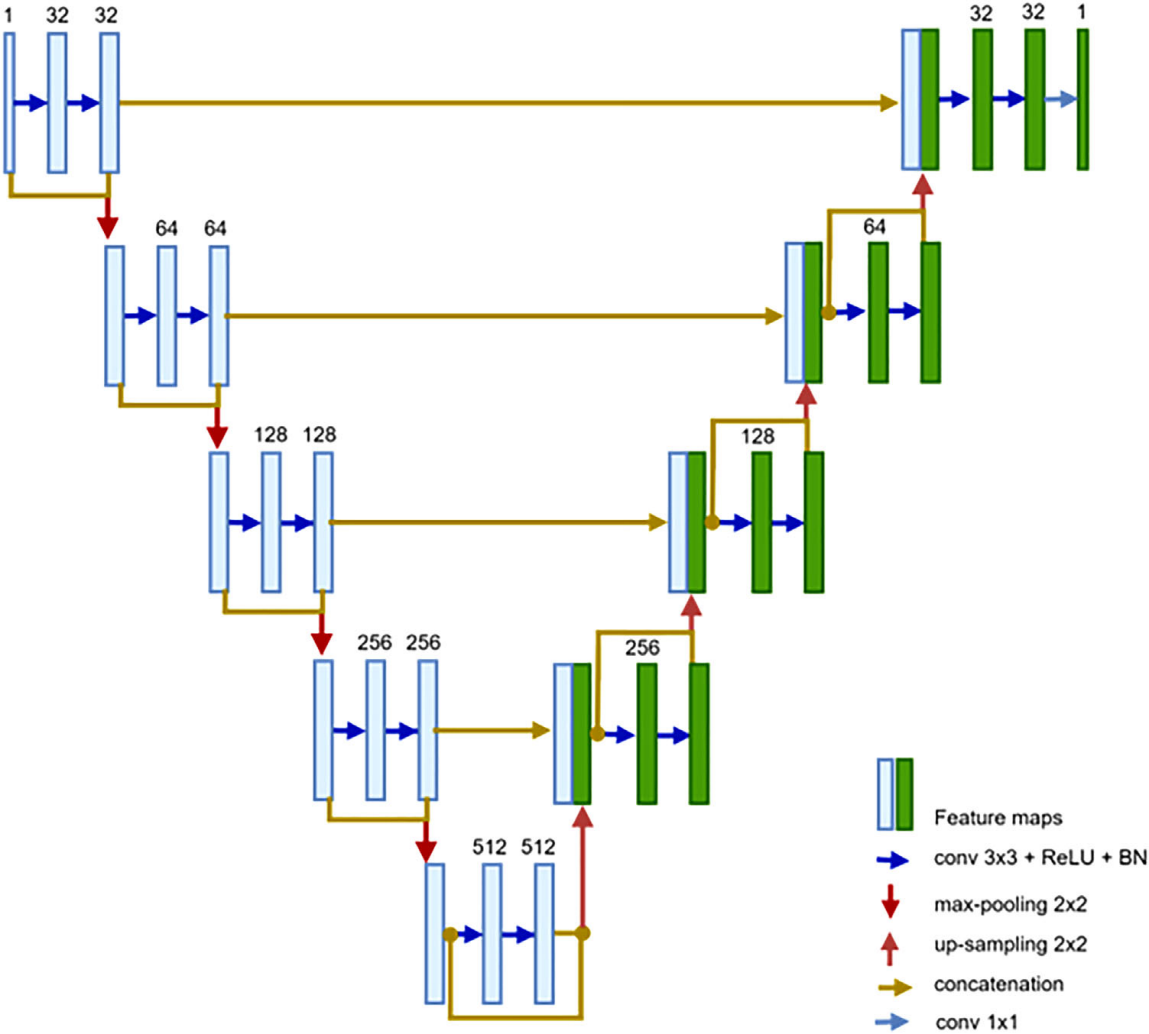
The proposed FCN.

The segmentation of the hepatic vessels in this study uses a deep FCN. The proposed FCN network is built on a modified version of the U‐net design,^25^ which has five levels as illustrated in Figure 3. An encoding path and a decoding path make up the U‐net. Three operations—convolution, activation function (ReLU), and batch normalization—are used at each level of encoding. Each level block has two consecutive deployments of these operations, which are then followed by a max‐pooling operation before going to the next level. For convolutions, the kernel size is 3 × 3, while for max pooling, it is 2 × 2. After each stage, the feature's resolution is divided in half. The network's decoding path restores the original input size by using the same set of operations (conv, ReLU, BN), but by substituting out max‐pooling for up‐sampling at each level. Each decoding level's input also receives the equivalent characteristic from the encoding path concatenated. With 1 × 1 convolution and sigmoid activation function, the decoding path's final level produces a binary prediction map after classifying the feature map using the dice coefficient metric.

**FIGURE 4:**
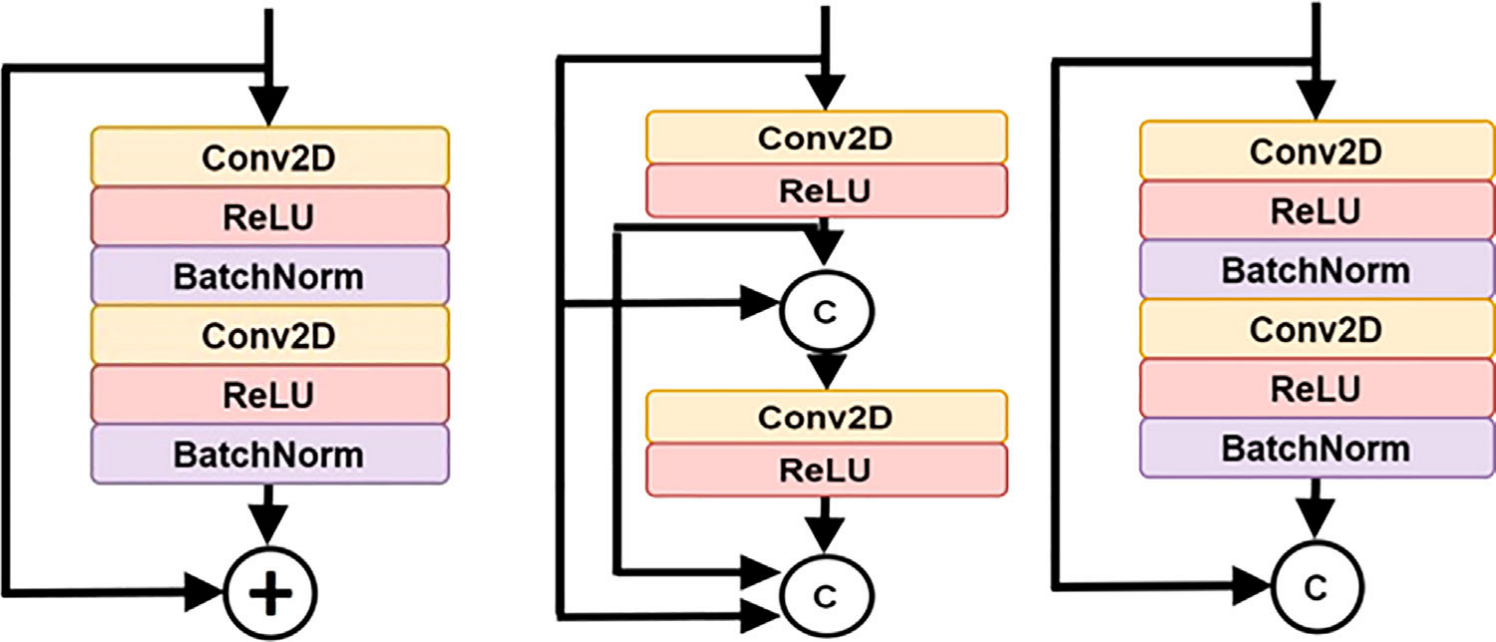
_(a) Residual block, (b) dense block, and (c) ResDense block._

The constant rise in network depth in FCN causes the vanishing gradient problem, which slows down training and worsens performance since additional layers are layered on top of each other and disappear. Although several deep network topologies were suggested to address this issue, DensNet and ResNet are considered major performance advances. Each layer in DensNet is connected to every forward layer, where the feature maps produced by various filter sizes are concatenated from earlier layers, significantly thickening the model as channels are merged following each convolution operation.^38^ But with ResNet, the prior input identity and output feature map are combined using the addition operation, so that does not happen. Figure 4 describes the differences in connections for (a) residual block and (b) dense block. In a ResNet block, a shortcut (skip connection) from the input of the block (identity) bypasses the stacked layers and attaches with the output feature of the block.^39^

By concatenating the features rather than summing them as in ResNet, DensNet tries to ensure that the most information can pass between network layers. As a result, DensNet is viewed as a memory‐hungry network since back‐propagation necessitates storing each layer's output, which uses up more memory and causes it to operate slowly. In contrast, the main focus of ResNet is the inclusion of tensors. However, it has been suggested that adding feature mappings directly degrades the gradient flow across the network because it sums up the values of the features.

Because the summation ruins the feature maps for both the convolution operation and the source of the skip connections, the concatenation operation is chosen because it preserves the feature maps. The ResDense block, as depicted in Figure 4c, is the primary contributor to the suggested network architecture. The suggested ResDense uses dense connections (concatenation) between residual blocks. The suggested ResDense block refines the feature values enough in terms of feature map flow and memory and intermittently remembers the refined feature values through dense connections between residual blocks.

The encoder and decoder routes of the proposed FCN network in this work make use of ResDense blocks. ResDense bricks are used to construct each level in the proposed network's expanding and contracting routes, as shown in Figure 4. As a result, at the end of each level in the encoding path, the depth size of the feature map is doubled and concatenated with block input.

### 2.5 Network implementation and training

The proposed network is trained using resized annotated 2D slices with patch sizes of 256 × 256 from cropped patches obtained from original sizes of 512 × 512, indicating that the training images are enlarged versions of the original images. Patch‐wise normalization of all CT slices was performed using zero mean and unit variance normalization.

To delineate the vascular areas within the liver ROI, a two‐redense network is constructed. Using the Adam optimizer, the network is trained, and the parameters are updated. The learning rate is set to start at 0.0001. The training process is observed using a variety of parameters. First, the learning rate is reduced by a factor of 0.2 if the validation loss does not improve after two consecutive epochs of training. The early termination of training, which occurs when the validation loss is not reduced during four successive epochs, is another monitoring parameter.

With a batch size of 32, the networks are trained. The soft dice coefficient loss is used to update the model's parameters and track the convergence of the network's training. The network architecture employs the batch normalization (BN) layer, which helps prevent the unreasonable growth or reduction of the generated values among network levels. The network's last layer makes use of the pixel‐wise sigmoid activation function.

Many initiatives have been taken to enhance the performance of the trained network due to the unbalanced class distribution of the vessel areas. The overlap between the ground‐truth patches and the region inside the liver ROI that the network has identified as vasculature is first measured during the training process using the soft dice loss metric. Additionally, the FCN network is only trained inside the liver ROI in order to learn features that only distinguish vessels from the parenchyma around the liver. In order to obtain the training patches, the CT slice's liver portion is cropped out entirely. Additionally, it is assured that the training patches employed have the corresponding mask and that patches without annotation mask are not included in the training process.

### 2.6 Performance measures

The segmentation of hepatic vessels from CT scans is assessed using a set of performance measures in order to quantitatively evaluate the proposed method. As indicated in Equation (1), the Dice coefficient (DSC), which is the first measure, computes the ratio between the successfully segmented class with regard to the average size between the segmentation output (A) and the ground truth (B). However, since we utilize the predicted probabilities directly rather than applying a threshold and turning them into a binary mask, the metric employed during the training phase is the soft Dice loss. We merely utilize 1 minus DSC value to formulate the loss function which can be minimized. A soft Dice loss is calculated for each class separately and then averaged to yield a final score.

(1) $$DSC=\frac{2\left|A\cap B\right|}{\left|A\right|+\left|B\right|}$$

Sensitivity is the second metric, which compares the proportion of correctly separated class voxels to the actual data. The method's ability to accurately segment the intended class of voxels is demonstrated by the sensitivity measure. The final metric, known as specificity, calculates the proportion of successfully segmented non‐class voxels to all non‐class voxels. The specificity metric demonstrates how likely it is for the method to segregate the voxels outside of the target class.

## 3 RESULTS AND DISCUSSION

**FIGURE 5:**
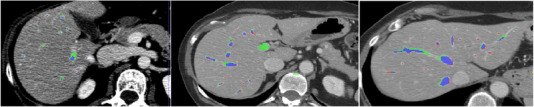
_Liver vasculature segmentation for different datasets._

The suggested method is assessed quantitatively and qualitatively on various test datasets from the Medical Segmentation Decathlon (MSD). Datasets are split into three groups with percentages of 80%, 10%, and 10% each: training, validation, and testing groups. The performance of the trained networks was remarkable. The segmentation of the liver vasculature using the suggested method is shown in Figure 5 for three separate data sets. The axial slices compare the output of the suggested approach with the equivalent segmentation of the ground truth. The segmented vessels mask is put over the CT scan in the areas of the figure where the procedure was successful in delineating the hepatic vasculature to show the results of the suggested method. By highlighting the key regions of the ground‐truth and the predicted segmentation, the axial slices explain the significant discrepancies between the segmentation technique output (green) and the manually delineated vasculature (red).

Visual inspection is used to conduct a qualitative evaluation of the results of the suggested method. The qualitative approach tries to highlight the key distinctions between the segmentation findings given in Figure 5 and the ground truth. The segmentation result's overlap with the ground truth is shown in blue. The vessels highlighted in green are over‐segmented (false positive) vessels, which are voxels segmented using the suggested procedure but absent from the ground truth. Voxels in the ground truth that the suggested approach was unable to segment are highlighted in red, signifying that the method under‐segmented them (false negative).

In general, it is evident that the procedure was successful in appropriately segmenting all of the major vessels. The terminals of the vessel branches and often the vessel surfaces are where the biggest variances occur. The majority of over‐segmentation cases, as shown in Figure 5, do not involve over‐segmented branches, but it is evident that the vessels are appropriately segmented by the proposed method even though it does not show up in the ground truth segmentation. The surfaces of the vessels and the tips of the branches are where most segmentation disparities between the segmented vasculature and its matching ground truth occur. The portal veins and the three major hepatic veins (right, center, and left) are well defined. Additionally, the majority of over‐segmentation incidents occur because these manual delineations are too conservative or neglect certain areas.

The suggested system performed well, according to quantitative evaluation, and it met performance measure targets at comparable levels. Table 1 shows the achieved segmentation measures values with and without the pre‐processing procedure to emphasize the impact of employing the two filters, vesselness and CED. With the filtering enhancement steps, the achieved Dice overlap is 79%, while for sensitivity and specificity, the scores were 82% and 95%, respectively. It is obvious that the filters enhanced the characteristics in the training CT scans, which improved the U‐net training process. The specificity in segmentation tasks for medical imaging refers to a model's capacity to recognize background classes in a dataset. In this study, the segmentation of the vessels class is concentrated in the liver ROI. To lessen the data imbalance, the training process is carried out using datasets from the liver ROI that have been cropped. However, Specificity scores close to 1 is standard and expected.

**TABLE 1:** Measures values of vessels segmentation

Task	DSC %	Sensitivity %	Specificity %
Vessels segmentation (without enhancement step)	68.00	61.00	96.00
Vessels segmentation (with enhancement step)	79.00	82.20	95.10

For several reasons, it is difficult to do quantitative comparisons with other segmentation approaches described in the literature.^13^, ^19^, ^31^, ^40^, ^41^, ^42^, ^43^, ^44^, ^45^, ^46^ First, there are not many studies on this topic of liver vasculature segmentation. Some authors used their own clinical data sets, others employed artificial data sets, and still others confirmed their findings using images of created forms (phantoms) resembling veins.^41^ Second, the public cannot access the implementations of other works. In addition, the majority of studies that suggested segmenting the liver vasculature used visual inspection to judge the accuracy of their segmentations. Some works used a small dataset like IRCAD datasets to train their deep learning network to segment vessels which affects its ability for generalization.^28^ other authors created a new Dense V‐Net (DV‐Net) by adding a dense block structure to the V‐net and utilize data augmentation with a small number of training samples to segment liver vessels from abdominal CT volumes. In addition, they suggest a D‐BCE loss function to make the most use of image resources and a dual‐branch dense connection down‐sampling approach (DCDS) to better capture vascular features. Authors compared their work with other unet based networks like 3D U‐net and 3D dense U‐net.^46^

Other study provided a region‐growing based method that is applied to a subset of IRCAD datasets. The authors did not provide a thorough evaluation of their method and employed a few performance metrics like dice or Jaccard metrics that do not accurately reflect the segmentation output accuracy. The accuracy and mistakes of the method as provided in this paper were only briefly evaluated in a few of these papers.^13^, ^41^, ^43^, ^45^ Results of the suggested method's evaluation are shown in Table 2 alongside those of other deep learning‐based techniques.

**TABLE 2:** Comparative evaluation

Method	DSC %	Sensitivity %	Specificity %
U‐net	70.43	70.89	98.77
Dense Unet	65.21	62.72	96.23
V‐net (Su et al.)	75.46	76.93	98.89
3D‐Unet (Huang et al.)	75.30	76.30	97.60
Proposed method	79.00	82.20	95.10

## 4 CONCLUSION

A deep learning‐based, totally automatic approach of segmenting hepatic arteries from CT scans was presented in the paper. The main contributions in this paper are the use of preprocessing filtering steps to enhance the training process of the U‐net based deep learning network. Also, the paper highlighted the ResDense block as one of the residual blocks that improve the learning step. The framework begins by applying two filters—vesselness and CED filters—to enhance the appearance of the vasculature in the training CT scans. The outcomes demonstrated how the preprocessing phase improved the U‐net network's learning process. The designed network made use of the U‐net architecture as its backbone, and each level of the proposed ResDense block‐based encoding and decoding pathways. Due to concatenation skip connections in each ResDense block, the feature maps of the vessels areas travel through the network without significantly changing their values, which increased network learning and improved segmentation performance. The proposed method worked successfully in properly segmenting the hepatic vasculature from all data sets. The inaccuracies that do occur in the findings are relatively small and mostly concentrated on the tips of the branches, so they have little impact on the treatment planning decision‐making process. The method's suitability as a clinical tool for preoperative planning in hepatic therapies is supported by the quantitative and qualitative evaluations.

## AUTHOR CONTRIBUTIONS

All authors have made substantial contributions to the analysis of the work, have helped draft and or edit the manuscript, provided approval of submission, and have worked to ensure the accuracy of the results presented.

## CONFLICT OF INTEREST STATEMENT

The authors declare no conflict of interest.

## ETHICS APPROVAL

All procedures performed in studies involving human participants were in accordance with the ethical standards of the institutional and/or national research committee and with the 1964 Helsinki declaration and its later amendments or comparable ethical standards. For this type of study, formal consent is not required.

## Data Availability

The data that support the findings of this study are openly available in The Medical Segmentation Decathlon (MSD) at http://medicaldecathlon.com/.
